# Advancements in understanding neuropsychiatric lupus: deciphering immune heterogeneity with single-cell resolution

**DOI:** 10.3389/fmed.2025.1732805

**Published:** 2026-01-13

**Authors:** Zhou Fan, Wang Hai, Zeng Xianyi, Xie Baozhao, Yan Jichun

**Affiliations:** 1 Department of General Practice, The First People's Hospital of Qinzhou, Qinzhou, China; 2 Department of Rheumatology and Immunology, Yue Bei People's Hospital, Shaoguan, China; 3 China Unicom Digital Intelligence Medical Technology Co, Ltd., Guangzhou, China; 4 Department of Rheumatology and Immunology, The Seventh Affiliated Hospital of Guangxi Medical University (Wuzhou Gongren Hospital), Wuzhou, China; 5 Ganzhou Hospital-Nanfang Hospital, Southern Medical University (Ganzhou People's Hospital), Ganzhou, Jiangxi, China

**Keywords:** diagnostic challenges, heterogeneous manifestations, neuropsychiatric lupus (NPSLE), pathogenic mechanisms, targeted therapies

## Abstract

Neuropsychiatric Systemic Lupus Erythematosus (NPSLE) represents one of the most severe and enigmatic manifestations of SLE, contributing significantly to disease morbidity and mortality. Its clinical presentation is profoundly heterogeneous, encompassing 19 distinct syndromes that pose considerable diagnostic and therapeutic challenges. While historical estimates of its prevalence varied widely, contemporary prospective studies suggest that approximately 56% of SLE patients experience a neuropsychiatric event, with 30–50% of these events being directly attributable to SLE-related pathogenic mechanisms. A comprehensive understanding of its multifactorial pathogenesis is critical for developing targeted therapies. The pathogenesis of NPSLE is an integrated neuroimmune process initiated by the disruption of the blood–brain and blood-CSF barriers. This breach permits peripheral autoimmune mediators-including pathogenic autoantibodies (e.g., anti-NMDAR, anti-ribosomal P), pro-inflammatory cytokines (e.g., Type I IFN, IL-6), and innate immune cells such as neutrophils forming Neutrophil Extracellular Traps (NETs)-to enter the central nervous system (CNS). Within the CNS, these insults trigger a robust response from resident glial cells. Microglia polarize to a pro-inflammatory M1 phenotype, driving neuroinflammation and pathological synaptic stripping, while astrocytes transition to a neurotoxic A1 phenotype, losing their neurosupportive functions. These inflammatory pathways are intricately linked with an ischemic/thrombotic pathway, often mediated by antiphospholipid antibodies, creating a complex pathogenic landscape. The adaptive immune system plays a crucial role in perpetuating the autoimmune attack within the CNS compartment. This involves the infiltration of pathogenic T cell subsets, as identified in animal models like the MRL/lpr strain, including T follicular helper (Tfh)-like cells in the choroid plexus and senescent/exhausted Eomes+ double-negative T cells within the brain parenchyma. Furthermore, the CNS can become an active site for B cell responses, supporting the intrathecal clonal expansion of B cells and plasma cells, leading to local production of pathogenic autoantibodies. Deconstructing this cellular and molecular complexity requires high-resolution technologies. Single-cell genomics (scRNA-seq, CITE-seq, scBCR-seq) and spatial transcriptomics are providing unprecedented insights into the specific cell types and states driving the disease. While direct analysis of human NPSLE brain tissue remains rare, a powerful framework is emerging from synthesizing mechanistic data from diverse animal models (e.g., MRL/lpr and NZB/W F1) with findings from human CSF in related neuroinflammatory conditions. The translation of these molecular insights into clinical practice is the ultimate goal. This research paves the way for moving beyond descriptive syndromic classifications toward a mechanism-based stratification using “immune endophenotypes.” Such an approach promises to enhance biomarker discovery (e.g., NfL, GFAP, CXCL13) and guide the development of targeted therapies, such as Type I IFN receptor blockade and specific B cell-directed agents. By bridging high-resolution discovery with clinical reality, we can advance toward an era of precision medicine for the management of NPSLE.

## 1 Introduction to neuropsychiatric systemic lupus erythematosus

### 1.1 The clinical challenge of SLE and its neuropsychiatric manifestations

Systemic Lupus Erythematosus (SLE) is a quintessential systemic autoimmune disease defined by a profound loss of self-tolerance, leading to the production of a wide array of autoantibodies and subsequent multi-organ inflammation and damage (1). Nervous system involvement represents one of the most severe and least understood challenges in the management of the disease.

The nomenclature used to describe this condition has evolved in parallel with our understanding of its pathology. Historically, terms such as “lupus cerebritis” or “lupus sclerosis” were employed, particularly when central nervous system (CNS) inflammation was presumed to be the primary driver (1). However, these terms are now considered outdated as they fail to capture the full mechanistic diversity of the condition. The modern, accepted term is Neuropsychiatric SLE (NPSLE), which serves as a broad umbrella for the entire spectrum of neurological and psychiatric syndromes affecting both the central and peripheral nervous systems (1). This terminological shift reflects a critical paradigm change-away from a singular focus on inflammation (“-itis”) and toward a more sophisticated appreciation of a multifactorial syndrome encompassing vascular, metabolic, and neurotoxic mechanisms that are not always overtly inflammatory. This review will use the term NPSLE throughout, specifying CNS or peripheral nervous system (PNS) involvement where appropriate. Regardless of nomenclature, NPSLE contributes significantly to disease-related morbidity, mortality, and a profoundly diminished quality of life for patients (2).

### 1.2 Epidemiology and attribution: a realistic appraisal of prevalence and impact

The true frequency of NPSLE has been a subject of considerable debate, with historical estimates varying dramatically from as low as 12% to as high as 95% (3). This wide range is largely a product of methodological differences between studies, including retrospective versus prospective designs, inconsistencies in case definitions, and the inclusion of both major and minor, non-specific syndromes (2). More recent, rigorous meta-analyses of prospective studies have provided a more realistic estimate, indicating that approximately 56% of systemic lupus erythematosus (SLE) patients will experience at least one neuropsychiatric event during their disease course. Among these events, common manifestations such as headache, mood disorders, and cognitive dysfunction are the most frequent contributors to this figure (4).

Critically, a neuropsychiatric event occurring in an SLE patient is not synonymous with NPSLE. The concept of attribution is paramount; rigorous clinical evaluation is required to determine whether a given event is a direct consequence of SLE-related pathogenic mechanisms or is attributable to other causes, such as medication side effects, infections, or comorbid conditions. In daily clinical practice, attribution remains largely a diagnosis of exclusion, typically relying on multidisciplinary consensus to rule out “mimics” (e.g., infections, metabolic derangements) rather than solely on research-based attribution algorithms. Large, prospective cohort studies have consistently shown that only a fraction of these events-estimated to be between 30 and 50%-can be directly attributed to SLE itself (5). This distinction is not merely academic; it is fundamental to appropriate management, as misattribution can lead to unnecessary and potentially harmful immunosuppression.

### 1.3 The heterogeneity of NPSLE: from ACR syndromes to pathogenic mechanisms

The clinical heterogeneity of NPSLE is formally captured by the 19 distinct syndromes defined by the American College of Rheumatology (ACR) in 1999, which are broadly divided into 12 affecting the CNS and seven affecting the PNS (2). These syndromes can be further categorized based on their clinical presentation as either focal or diffuse (2). Focal manifestations, such as stroke, transverse myelitis, or seizures, are characterized by neurological deficits that can be localized to a specific neuroanatomical area. In contrast, diffuse manifestations, including cognitive dysfunction, psychosis, mood disorders, and acute confusional states, involve more widespread brain dysfunction.

This clinical distinction provides a foundational framework for considering the underlying pathophysiology, which is broadly divisible into two major mechanistic pathways: ischemic/thrombotic and autoimmune/inflammatory (6). Ischemic events are frequently driven by a prothrombotic state, often in the context of antiphospholipid antibodies (aPL), leading to vasculopathy and thrombosis. The inflammatory pathway is more complex, involving the direct or indirect effects of autoantibodies, pro-inflammatory cytokines, and infiltrating immune cells on neuronal and glial function. It is now clear that these pathways are not mutually exclusive and often interact, creating a complex pathogenic landscape that underlies the diverse clinical presentations of NPSLE and poses significant diagnostic and therapeutic challenges (7). Table 1 provides an overview of key NPSLE syndromes and their primary pathogenic considerations.

**Table 1:** Key neuropsychiatric syndromes in SLE and their primary pathogenic considerations.

NPSLE syndrome	Common clinical features	Predominant proposed pathogenesis	Key associated autoantibodies/biomarkers	References
Psychosis	Hallucinations, delusions, thought disorder	Inflammatory/Autoantibody-mediated	Anti-Ribosomal P, Anti-NMDAR (NR2 subunit), IFN-α in CSF, IL-6	(2, 3, 20, 21)
Seizures	Generalized or focal; convulsions, altered consciousness	Inflammatory/Autoantibody-mediated, Ischemic	aPL, Anti-MAP-2, CSF IL-6	(24, 47)
Cognitive dysfunction	Impairment in memory, attention, executive function, processing speed	Diffuse Inflammatory/Autoantibody-mediated, Microvascular	Anti-NMDAR, Anti-Ribosomal P, various cytokines (e.g., IL-6, TNF-α), aPL	(21, 24, 47, 48)
Stroke/TIA	Focal neurological deficits (weakness, sensory loss, aphasia, visual changes)	Ischemic/Thrombotic (often aPL-mediated)	Antiphospholipid antibodies (aPL) (Lupus Anticoagulant, anti-Cardiolipin, anti-β2GPI)	(3)
Aseptic meningitis	Headache, fever, nuchal rigidity, CSF pleocytosis (non-infectious)	Inflammatory	Elevated CSF proteins/cells	(7)
Demyelinating syndrome	Optic neuritis, transverse myelitis, MS-like lesions; vision loss, weakness, sensory loss	Inflammatory/Autoantibody-mediated	Anti-AQP4 (NMO-IgG) in some cases, other anti-myelin or anti-neuronal antibodies	(49)
Acute confusional state	Delirium; altered consciousness, attention deficits, disorganized thinking	Diffuse Inflammatory/Metabolic	Elevated CSF IL-6, Q albumin; often associated with high systemic disease activity	(50)
Mood disorder (depression/anxiety)	Persistent sadness, anhedonia, worry, irritability	Diffuse Inflammatory/Neurochemical/Psychological	Anti-Ribosomal P (depression), various cytokines; often multifactorial	(51)

## 2 The multifactorial pathogenesis of NPSLE: an integrated view

**Figure 1:**
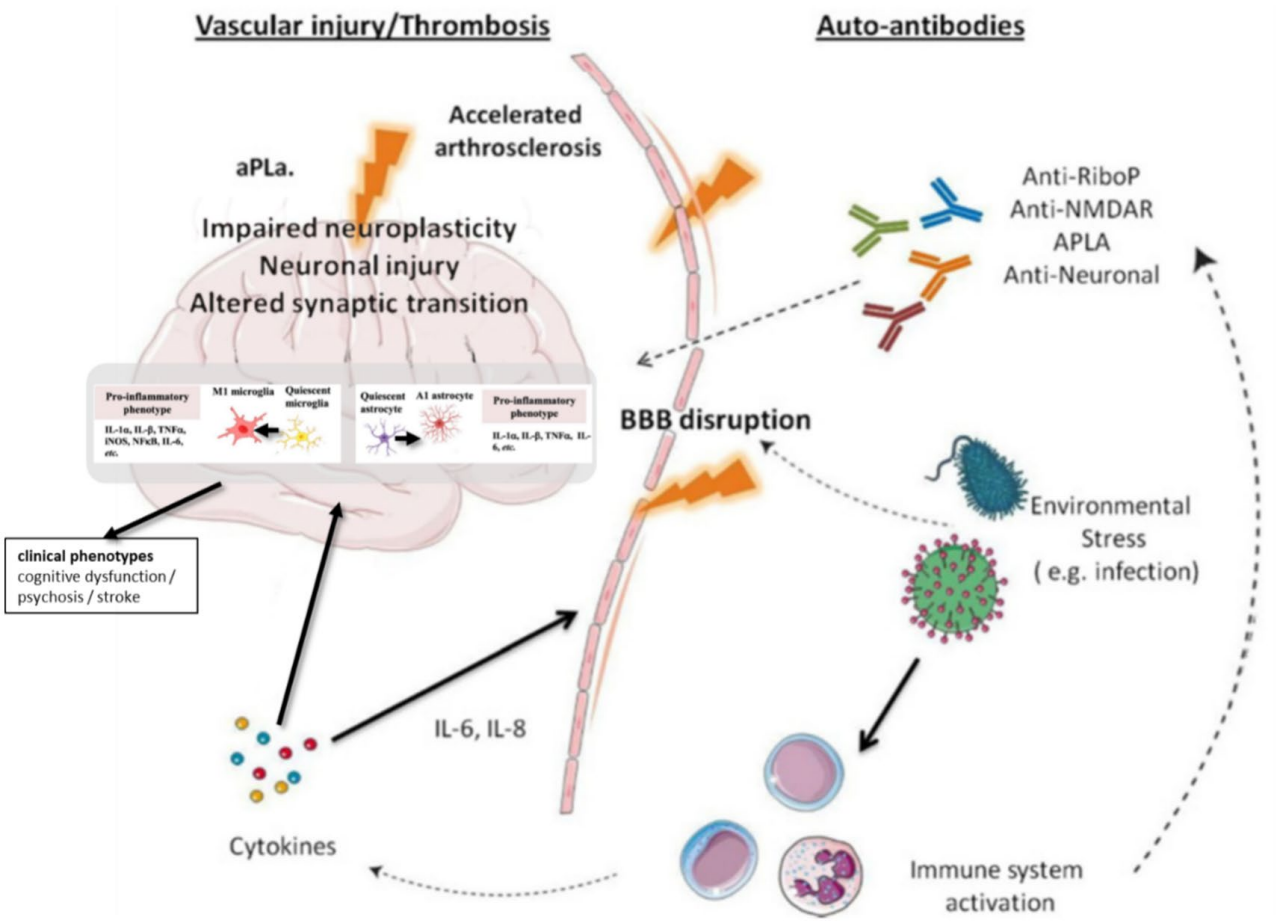
The integrated pathogenesis of neuropsychiatric systemic lupus erythematosus (NPSLE).

The pathogenesis of NPSLE is not simply a peripheral immune attack on a passive CNS. Rather, it is a dynamic, bidirectional process involving a compromised neurovascular unit, an actively responding resident CNS immune system, and a sustained assault from peripheral autoimmune mediators. This integrated view reframes NPSLE as a true neuroimmune disorder, where interactions between the brain and the systemic immune system are central to the disease process (Figure 1).

The pathogenesis of NPSLE is not simply a peripheral immune attack on a passive CNS. Rather, it is a dynamic, bidirectional process involving a compromised neurovascular unit, an actively responding resident CNS immune system, and a sustained assault from peripheral autoimmune mediators. This integrated view reframes NPSLE as a true neuroimmune disorder, where interactions between the brain and the systemic immune system are central to the disease process.

### 2.1 Breaching the fortress: the central role of blood–brain and blood-CSF barrier disruption

The CNS is protected by highly specialized barriers, primarily the blood–brain barrier (BBB) formed by tight junctions between endothelial cells, and the blood-cerebrospinal fluid (CSF) barrier located at the choroid plexus (8). Disruption of these barriers is a critical initiating event in many forms of NPSLE, as it permits the entry of circulating autoantibodies, inflammatory cytokines, and immune cells from the periphery into the brain microenvironment (8).

Evidence for BBB permeability in NPSLE comes from multiple sources. Analysis of CSF often reveals an elevated albumin quotient (Qalb), which indicates leakage of this high-molecular-weight serum protein into the CNS (9). More advanced neuroimaging techniques, such as dynamic contrast-enhanced magnetic resonance imaging (DCE-MRI), provide a quantitative measure of this disruption. Studies using DCE-MRI have demonstrated increased permeability, reflected by a higher volume transfer constant (Ktrans), particularly in the hippocampus of SLE patients, even in the absence of overt neuropsychiatric symptoms (8). The integrity of these barriers can be compromised by systemic inflammation, the direct action of cytokines like IL-6, and autoantibody-mediated endothelial injury, creating a gateway for the peripheral immune system to access and damage the CNS (9).

### 2.2 The brain’s own immune response: microglia and astrocytes as key drivers of neuroinflammation

Once the BBB is breached, the resident immune cells of the CNS-microglia and astrocytes-are not passive bystanders. They become key amplifiers and drivers of the neuroinflammatory cascade, shaping the nature and severity of the neurological deficit.

Microglia, the brain’s resident macrophages, are exquisitely sensitive to disturbances in CNS homeostasis. In NPSLE, they undergo a profound activation, polarizing towards a pro-inflammatory M1 phenotype (10). This is evidenced by increased levels of M1-associated cytokines (e.g., IL-1, IL-6, TNF-*α*) in the CSF of NPSLE patients and in the brains of MRL/lpr lupus mice (10). A key intracellular platform for this pro-inflammatory response is the NLRP3 inflammasome, which, upon activation in microglia, processes pro-IL-1 into its highly inflammatory mature form (11). Transcriptomic analysis of microglia from lupus models reveals the upregulation of genes associated not only with inflammation but also with phagocytosis (12–15). This enhanced phagocytic activity leads to a pathological process known as synaptic stripping, where activated microglia engulf and eliminate synapses, a mechanism directly implicated in the cognitive deficits and anxiety-like behaviors observed in animal models of NPSLE (10).

Astrocytes, the most abundant glial cells in the CNS, also play a dual role (16). Under pathological conditions like NPSLE, they can become reactive and adopt different phenotypes. Pro-inflammatory signals from activated M1 microglia, such as IL-1, TNF-*α*, and C1q, can induce astrocytes to polarize into a neurotoxic A1 phenotype. A1 astrocytes lose their normal neurosupportive functions and release factors that are directly toxic to neurons and oligodendrocytes (17). Evidence of astrocytic injury in NPSLE is robust, primarily through the measurement of glial fibrillary acidic protein (GFAP), an astrocyte-specific intermediate filament protein. Elevated levels of GFAP in the CSF of NPSLE patients are a reliable marker of astrocytic damage and correlate with disease activity and abnormalities on conventional MRI (18). More recently, serum GFAP has emerged as a promising, less invasive biomarker that is also elevated in active major NPSLE, reflecting this ongoing glial pathology (19).

### 2.3 The peripheral assault: autoantibodies, cytokines, and the innate immune system

The inflammatory cascade within the CNS is initiated and sustained by a continuous influx of pathogenic mediators from the systemic circulation.

Autoantibodies are central to this process. Several specific autoantibodies have been strongly implicated in NPSLE through direct pathogenic mechanisms:

- Anti-N-methyl-D-aspartate receptor (NMDAR) antibodies, a subset of anti-dsDNA antibodies that cross-react with the NR2 subunit of the NMDAR, can cause excitotoxic neuronal death upon entering the brain, leading to cognitive and behavioral deficits (20).
- Anti-ribosomal P protein antibodies have a controversial but persistent association with lupus psychosis and depression. Evidence suggests they can bind to a neuronal surface protein, triggering calcium influx and apoptosis, and may directly contribute to memory impairment (21).
- Antiphospholipid antibodies (aPL) are the primary mediators of the ischemic pathway in NPSLE, promoting a prothrombotic state that leads to stroke and transient ischemic attacks. However, emerging evidence also suggests they may exert direct, non-thrombotic effects on neuronal cells (22).

Pro-inflammatory cytokines produced systemically can cross a compromised BBB or be produced intrathecally, further driving neuroinflammation:

- Type I Interferons (IFN-*α*) are a hallmark of SLE pathogenesis, creating a systemic “IFN signature.” In the context of NPSLE, IFN-α is a potent activator of microglia, can induce synaptic pruning, and is strongly associated with the development of psychosis (3).
- Interleukin-6 (IL-6) is another key cytokine whose levels are markedly elevated in the CSF of patients with active NPSLE, particularly in those with acute confusional states. IL-6 can promote inflammation through both classic and trans-signaling pathways and can contribute to BBB dysfunction (1).

Neutrophils, key cells of the innate immune system, also contribute significantly. In SLE, neutrophils are prone to forming Neutrophil Extracellular Traps (NETs), web-like structures of decondensed chromatin and granular proteins. These NETs are a potent source of autoantigens (e.g., dsDNA, histones) and pro-inflammatory mediators (e.g., neutrophil elastase). By promoting endothelial cell damage and thrombosis, NETs can contribute to both BBB disruption and the ischemic manifestations of NPSLE (23).

### 2.4 Ischemic vs. inflammatory pathways: an integrated perspective

The traditional dichotomy between ischemic and inflammatory NPSLE is an oversimplification that fails to capture the intricate interplay between these pathways. Systemic inflammation, driven by cytokines and activated innate immune cells, creates a prothrombotic state by activating endothelial cells and platelets (15). Conversely, vascular damage and ischemia can further disrupt the BBB, creating a feed-forward loop that facilitates the entry of more inflammatory mediators and immune cells into the CNS (6). This integration of mechanisms explains why some patients present with “mixed” phenotypes and underscores the need for therapies that can address both arms of NPSLE pathogenesis. Table 2 summarizes the key immune cell populations involved in this integrated model.

**Table 2:** Key immune cell populations in NPSLE pathogenesis.

Cell type	Key markers/features	Location (CNS/periphery)	Proposed pathogenic role	References
M1 microglia	CD86, iNOS, NLRP3 inflammasome activation, IL-1 production	CNS (resident)	Neuroinflammation, synaptic stripping, neuronal death	(10)
A1 astrocytes	C3 expression, GFAP release	CNS (resident)	Neurotoxic factor release, loss of neurosupportive function, neuronal death	(38)
Neutrophils	NET formation, Myeloperoxidase (MPO), Neutrophil Elastase	Periphery → CNS	Endothelial damage, BBB disruption, thrombosis, source of autoantigens	(52)
CD4 + T follicular helper (Tfh)-like cells	Bcl-6, IL-21, IFN-γ, ICOS+, PD-1+	Periphery → Choroid Plexus	B cell help, promotion of intrathecal autoantibody production, local inflammation	(53)
Th17 cells	IL-17 production	Periphery → CNS	Pro-inflammatory cytokine production, BBB disruption, tissue inflammation	(25)
Eomes+ double-negative T cells (DNTs)	Eomes, CD3+, CD4-, CD8-	Periphery → Brain Parenchyma	Cellular senescence/exhaustion, interaction with glia, neuroinflammation	(7)
Intrathecal plasma cells	CD138+, CD27high, IgG1 production	CNS (intrathecal)	Local production of pathogenic autoantibodies, perpetuation of CNS inflammation	(26)
Age-Associated B Cells (ABCs)	T-bet+, CD11c+	Periphery	Precursors to autoantibody-secreting cells, pro-inflammatory cytokine production	(27)

## 3 The adaptive immune response in the CNS compartment

While the innate immune system and resident glial cells establish the neuroinflammatory milieu, the adaptive immune system-comprising T and B lymphocytes-provides the specificity and sustained drive for the autoimmune attack within the CNS.

### 3.1 T cell heterogeneity in NPSLE: pathogenic effectors, regulatory imbalance, and exhaustion

T lymphocytes are critical orchestrators of the autoimmune response in SLE, and their infiltration into the CNS is a key feature of inflammatory NPSLE (7). A fundamental aspect of this pathology is an imbalance between effector and regulatory T cell populations.

Regulatory T cells (Tregs), which are essential for maintaining self-tolerance, are often numerically deficient or functionally impaired in SLE, allowing autoreactive T cells to escape control and proliferate (24). Concurrently, pro-inflammatory T helper subsets, including IFN-*γ*-producing Th1 cells and IL-17-producing Th17 cells, are expanded and contribute to tissue damage and BBB breakdown (25).

Animal models have been instrumental in dissecting the heterogeneity of pathogenic T cells within the CNS. In the MRL/lpr mouse model, two distinct populations have been identified as key contributors:

1. T follicular helper (Tfh)-like cells: A unique subset of CD4 + T cells expressing the transcription factor Bcl-6 and producing high levels of IL-21 and IFN-*γ* has been found to accumulate specifically in the choroid plexus. This strategic location at the blood-CSF barrier suggests they play a pivotal role in providing “help” to B cells, thereby orchestrating a local, intrathecal autoantibody response (7).
2. Eomes+ Double-Negative T cells (DNTs): A population of T cells lacking both CD4 and CD8 co-receptors but expressing the transcription factor Eomesodermin has been identified within the brain parenchyma. These cells display transcriptional signatures of cellular senescence and exhaustion, a state of dysfunction arising from chronic antigen stimulation. Their interactions with glial cells suggest a role in perpetuating a state of chronic, low-grade neuroinflammation (1). This theme of T cell exhaustion is not unique to the mouse model; it is also observed in clonally expanded T cells within the human CSF in other chronic neuroinflammatory conditions, suggesting it may be a common pathway for T cells responding to persistent inflammation within the CNS (26).

### 3.2 B cells and intrathecal antibody production: clonal expansion and local niches

The presence of autoantibodies in the CSF of NPSLE patients has long implicated B cells in the disease process. It is now increasingly clear that the CNS is not merely a passive recipient of peripherally produced antibodies. Instead, it can become an active site of B cell responses, supporting local differentiation, clonal expansion, and intrathecal antibody production (1).

Studies of CSF from patients with various neuroinflammatory disorders have consistently shown an enrichment of B cells and, most notably, antibody-secreting cells (ASCs) like plasma cells and plasmablasts (26). These intrathecal B cell populations are often clonally expanded and have undergone immunoglobulin class-switching and somatic hypermutation, hallmarks of a mature, antigen-driven immune response occurring within the CNS compartment (26). This local immune activity is fostered by a supportive microenvironment, which includes the production of the potent B cell chemoattractant CXCL13 and B cell survival factors such as BAFF and APRIL (27). Pathogenic B cell subsets that are expanded systemically in SLE, such as Age-Associated B Cells (ABCs), are also likely contributors to the pool of autoreactive cells that can enter and function within the CNS (28).

Flow cytometric studies of CSF from patients with neuropsychiatric connective tissue diseases (N-CTD), including NPSLE, have provided important diagnostic context. While one key study by Heming et al. found no single CSF parameter that could reliably distinguish N-CTD from non-neuropsychiatric CTD, it made a crucial observation: an expansion of plasma cells in the peripheral blood was highly effective at differentiating N-CTD patients from those with multiple sclerosis (MS) (29). This highlights that while intrathecal B cell activity is a feature of neuroinflammation, specific signatures in the peripheral blood may hold greater differential diagnostic power, reflecting the systemic nature of the disease driving CNS pathology.

### 3.3 Insights from animal models: contrasting the MRL/lpr and NZB/W F1 strains

Spontaneous mouse models of lupus have been invaluable for mechanistic studies, though it is crucial to recognize that no single model perfectly recapitulates the human disease. A balanced perspective requires considering insights from multiple strains.

The MRL/lpr mouse is perhaps the most widely studied model for NPSLE. These mice develop an aggressive, accelerated autoimmune disease due to a mutation in the *Fas* gene, leading to defective lymphocyte apoptosis. They exhibit a prominent neurobehavioral phenotype, including depression- and anxiety-like behaviors and cognitive deficits, which often appear early in the disease course (30). As discussed, their CNS pathology is characterized by early and robust T cell infiltration into the choroid plexus and widespread microglial activation (10).

The New Zealand Black/White (NZB/W) F1 mouse represents another key spontaneous model. In contrast to MRL/lpr mice, NZB/W F1 mice develop a more indolent disease with a later onset of neuropsychiatric symptoms (31). Their behavioral phenotype is also distinct, primarily characterized by impaired learning and memory and increased anxiety, rather than the prominent depressive-like features of the MRL/lpr strain (31). The neuropathology in NZB/W F1 mice often involves diffuse lymphoproliferative infiltrates in the meninges, choroid plexus, and perivascular spaces, along with evidence of immune complex deposition (32).

The differences between these models are instructive. The MRL/lpr strain, with its acute, T-cell-driven inflammation at the blood-CSF barrier, may better model the acute inflammatory or psychotic syndromes seen in human NPSLE. In contrast, the NZB/W F1 model, with its later onset and more diffuse, chronic immune infiltration, might be more representative of the progressive cognitive decline associated with widespread, smoldering neuroinflammation. Critically evaluating findings from both models provides a more comprehensive picture of the potential pathogenic pathways active in human disease (30). However, caution is warranted when extrapolating these findings to human disease, as murine models often possess specific genetic mutations (e.g., Fas) and display simplified behavioral phenotypes that do not fully capture the complexity of human NPSLE.

## 4 Unraveling cellular complexity with high-resolution technologies

The profound cellular heterogeneity underlying NPSLE pathogenesis necessitates the use of high-resolution technologies that can dissect immune responses at the single-cell level. These approaches are beginning to move the field beyond bulk measurements toward a granular understanding of which specific cell types and states drive distinct aspects of the disease.

### 4.1 Applying single-cell genomics to decipher immune contributions in NPSLE

Single-cell RNA sequencing (scRNA-seq) has revolutionized immunology by enabling the transcriptomic profiling of thousands of individual cells simultaneously, revealing cellular identity, activation states, and functional potential (33). Multimodal approaches like Cellular Indexing of Transcriptomes and Epitopes by Sequencing (CITE-seq) further enhance this by concurrently measuring surface protein expression, providing a more robust classification of immune cell subsets (34). These technologies are indispensable for deconstructing a clinically and pathologically complex disease like NPSLE (7).

However, it is crucial to acknowledge the current landscape of this research accurately. Direct scRNA-seq studies on brain tissue or CSF from human NPSLE patients are exceptionally rare due to the invasive nature of sample collection. Therefore, our current high-resolution understanding is a mosaic, built from detailed mechanistic insights from animal models and inferences drawn from human CSF studies in related, and often more common, neuroinflammatory diseases like MS (26). Synthesizing these disparate data sources allows for the generation of robust, testable hypotheses for NPSLE.

### 4.2 Single-cell signatures of T cell subsets in neuroinflammation

The application of scRNA-seq to the MRL/lpr mouse brain has provided a detailed catalog of the T cell landscape in lupus-associated neuroinflammation, identifying the senescent/exhausted Eomes+ DNTs and other conventional T cell subsets (7). To understand the potential relevance of these findings to human disease, we can turn to large-scale single-cell atlases of human CSF. These studies, while not specific to NPSLE, reveal common features of T cell responses during neuroinflammation. They consistently show that CNS-infiltrating T cells shift towards memory and effector phenotypes. Clonally expanded T cells, often cytotoxic CD8 + subsets, upregulate genes associated with cytotoxicity (e.g., granzymes, perforin), CNS trafficking (e.g., CXCR3), and markers of senescence or exhaustion (26). This convergence of findings-particularly the theme of T cell exhaustion in both the lupus mouse brain and inflamed human CSF-suggests that this may be a shared terminal differentiation state for T cells operating within the chronic inflammatory milieu of the CNS.

### 4.3 Dissecting B cell clonal evolution and function with scRNA-seq and scBCR-seq

A particularly powerful application of single-cell technology is the combination of scRNA-seq with single-cell B cell receptor sequencing (scBCR-seq). This dual-omics approach makes it possible to directly link a B cell’s complete transcriptional profile-revealing its activation state, functional potential, and interaction pathways-to the precise sequence of its B cell receptor (35).

For NPSLE, this technology holds transformative potential. By applying it to B cells isolated from the CSF, researchers could definitively identify the antigen specificities of the autoreactive B cell clones operating within the CNS. Furthermore, it would enable the tracking of clonal lineages between the CSF and peripheral blood, clarifying trafficking patterns and cellular origins. This approach could ultimately reveal whether distinct NPSLE clinical subtypes (e.g., psychosis vs. cognitive dysfunction) are driven by unique CNS B cell receptor repertoires or are associated with specific B cell functional states, thereby pinpointing the “culprit” clones and their pathogenic mechanisms (36).

### 4.4 Mapping the neuroinflammatory landscape with spatial transcriptomics

A key limitation of standard scRNA-seq is that the process of tissue dissociation results in the loss of all spatial context. Spatial transcriptomics is an emerging technology that overcomes this by measuring gene expression within the intact tissue architecture (7). This allows identified cell types and states to be mapped back to their precise locations, revealing cellular neighborhoods and interactions.

Recent pioneering studies have applied this technology to the brains of lupus mouse models. These investigations have revealed that the Type I IFN gene signature, a key pathogenic driver in NPSLE, is not uniformly distributed but is enriched in spatially distinct patches within the brain parenchyma, particularly in subcortical areas (37). This finding provides strong evidence against a model of passive diffusion of peripheral IFN into the brain and instead points toward localized production or response. By integrating scRNA-seq data with these spatial maps, it becomes possible to understand how different cell types (e.g., IFN-producing glial cells and IFN-responding neurons) are organized and interact within these inflammatory foci, providing unprecedented insight into the structural basis of neuroinflammation in NPSLE. Table 3 summarizes the key findings from these and other relevant high-parameter studies, providing a comparative overview of the insights gained from different models and patient cohorts.

**Table 3:** Insights from single-cell and high-parameter studies in NPSLE and relevant models.

Model/patient cohort	Technology used	Key T cell findings	Key B cell findings	Relevance/limitations for NPSLE	References
MRL/lpr mice (brain, spleen)	scRNA-seq	Identified Eomes+ DNTs (senescent/exhausted, interact with glia); also CD4+, CD8+, Tregs.	B cells and plasma cells identified in brain; proportions differ from controls.	*Relevance:* Highlights specific pathogenic T cell subset (DNTs) and confirms B cell presence in a key lupus model. *Limitation:* Animal model may not fully recapitulate human disease.	(7)
MRL/lpr mice (choroid plexus, brain)	Multiparameter flow cytometry	Identified activated effector CD4 + T cells; pathogenic Tfh-like subset (IL-21+, IFN-γ+, Bcl-6+); diminished local Tregs.	Not the primary focus, but Tfh presence implies local B cell interaction.	*Relevance:* Pinpoints the choroid plexus as a key entry site and identifies pathogenic Tfh cells as drivers of neurobehavioral deficits. *Limitation:* Flow cytometry provides protein-level data, not full transcriptome.	(53)
Human CSF (MS, Other Inflammatory Neurological Disorders, Infectious, Non-inflammatory)	scRNA-seq, lymphocyte receptor sequencing	T cells shift to memory/effector phenotypes; increased Tfh in inflammatory cohorts; clonal expansion of cytotoxic/senescent T cells.	CSF enriched with clonally expanded, class-switched B cells & ASCs; shared transcriptional signatures in expanded memory B cells (e.g., *SUB1*).	*Relevance:* Provides a general landscape of human intrathecal inflammation, showing common patterns of T/B cell clonal expansion and activation potentially relevant to inflammatory NPSLE. *Limitation:* Not specific to NPSLE; findings are analogous.	(32)
Human N-CTD (incl. NPSLE), CTD, MS, Controls (CSF, blood)	Flow cytometry	CSF: CD4+/CD8 + ratio lower in N-CTD vs. MS. Blood: CD4 + T cells & CD4+/CD8 + ratio elevated in N-CTD vs. CTD.	CSF: Increased B cells and plasma cells in N-CTD vs. controls. Blood: Plasma cells expanded in N-CTD; useful for differentiating N-CTD from MS.	*Relevance:* Suggests intrathecal B cell expansion in N-CTD and highlights the diagnostic utility of peripheral (blood) immune profiles. *Limitation:* Lower resolution than sequencing; CSF shows no significant difference between N-CTD and non-neuro CTD.	(29)

## 5 Bridging molecular insights with clinical reality

The ultimate goal of applying high-resolution technologies to NPSLE is to translate molecular and cellular discoveries into tangible clinical benefits. This involves using these detailed insights to refine diagnostics, stratify patients more effectively, and develop targeted therapies that address the specific pathogenic mechanisms driving an individual’s disease.

### 5.1 Towards immune endophenotypes: linking cellular signatures to NPSLE subtypes

The current ACR classification system for NPSLE is descriptive and does not always align with underlying biology. Single-cell data offers the potential to move beyond this syndromic classification towards a more precise, mechanism-based stratification based on “immune endophenotypes” (distinct biological subgroups defined by specific immune mechanisms rather than just clinical symptoms). These endophenotypes would be defined by specific T and B cell transcriptional signatures, surface marker profiles, or unique immune receptor repertoires identified in CSF or blood. Such a biologically grounded re-classification could align more closely with pathogenic mechanisms. For example, one might hypothesize the existence of:

- An “IFN-Glial Driven Psychosis” endophenotype, characterized by a strong Type I IFN signature in CSF glial cells [as suggested by spatial transcriptomics in mice (37)] and the presence of intrathecally-derived anti-Ribosomal P antibodies produced by specific plasma cell clones (21).
- A “Microglia-Mediated Cognitive Dysfunction” endophenotype, marked by CSF biomarkers of microglial M1 activation and synaptic stripping, perhaps in conjunction with senescent/exhausted T cell populations or low-titer anti-NMDAR antibodies (7).
- A “Paucicellular Vasculopathic” endophenotype, associated with ischemic events, which would exhibit a less pronounced inflammatory cell signature in the CSF but might be defined by serum markers of endothelial activation, neutrophil activity, and high-titer aPL (38).

It is important to emphasize that these “immune endophenotypes” are currently hypothetical constructs derived largely from animal models. Their clinical validity remains to be established through “reverse translation” studies—for instance, by correlating single-cell profiles from patient biospecimens with specific clinical syndromes—to determine if they represent actionable clinical entities.

### 5.2 Implications for biomarker discovery: from CSF proteomics to advanced neuroimaging

There is a critical and unmet need for reliable biomarkers to improve the diagnosis of NPSLE, which currently relies on a combination of clinical judgment and the exclusion of other conditions (19).

Fluid biomarkers from CSF and blood are a major focus of this effort. Markers of neuronal and glial injury, such as neurofilament light chain (NfL) and GFAP, have shown significant promise. Studies have demonstrated that both CSF and serum levels of NfL and GFAP are elevated in patients with active major NPSLE, and serum NfL in particular shows excellent discriminatory power in distinguishing NPSLE from non-NPSLE controls (19). Other promising candidates include the B cell chemoattractant CXCL13, which is elevated in the CSF during neuroinflammation and may reflect intrathecal B cell activity, and novel markers identified through unbiased proteomics screens of CSF (39). However, widespread clinical adoption faces practical hurdles. While potentially discriminatory, these markers currently lack standardized commercial assays with defined, age-adjusted cutoffs for SLE populations. Furthermore, as markers of general neuronal injury rather than specific lupus pathology, they must be interpreted cautiously alongside clinical features.

Advanced neuroimaging offers a non-invasive window into brain pathology. Beyond conventional MRI, which is often normal or shows non-specific findings, advanced techniques can detect subtle microstructural and functional changes:

- Diffusion Tensor Imaging (DTI) can quantify the integrity of white matter tracts, often revealing reduced fractional anisotropy (FA) and increased mean diffusivity (MD) in NPSLE patients, indicative of microstructural damage (40).
- Magnetic Resonance Spectroscopy (MRS) measures the concentration of brain metabolites, such as N-acetylaspartate (a marker of neuronal health), providing a biochemical snapshot of neuronal function and injury (41).
- Positron Emission Tomography (PET), using radioligands that bind to targets like the translocator protein (TSPO), can visualize and quantify neuroinflammation, particularly microglial activation *in vivo* (42).

### 5.3 Future therapeutic avenues: targeting specific cells and pathways

A more granular understanding of NPSLE pathogenesis is essential for moving beyond broad immunosuppression towards targeted therapies (43). While conventional immunosuppressants like cyclophosphamide and mycophenolate mofetil (MMF) remain important for severe, inflammatory NPSLE, their use is based on broad efficacy in SLE rather than specific evidence in NPSLE (44).

The advent of biologic therapies offers the potential to target specific pathogenic pathways identified in NPSLE:

- B cell-directed therapies, such as the B cell-depleting agent rituximab and the BAFF inhibitor belimumab, are logical choices given the role of B cells and autoantibodies. While case series support the use of rituximab in refractory NPSLE, large clinical trials of biologics have often excluded patients with severe, active CNS disease, leaving their efficacy in this population an open question (45).
- Type I IFN receptor blockade with anifrolumab is a highly promising strategy, given the central role of the IFN signature in both systemic SLE and NPSLE pathogenesis. As with other biologics, dedicated trials are needed to confirm its efficacy for neuropsychiatric manifestations (46).

A significant hurdle in validating these targeted therapies is the historical exclusion of patients with active, severe CNS involvement from major randomized clinical trials, resulting in a scarcity of high-level evidence for this specific population. Future therapeutic development could target even more specific mechanisms highlighted by recent research, such as inhibitors of microglial activation, antagonists of the IL-6 receptor or IL-6 trans-signaling, or agents that selectively target pathogenic T cell subsets or restore Treg function.

## 6 Conclusion and outlook

Neuropsychiatric SLE remains one of the most formidable challenges in rheumatology, characterized by profound clinical heterogeneity and a complex, multifactorial pathogenesis. Single-cell sequencing and other high-resolution technologies are beginning to deconstruct this complexity, moving the field beyond broad concepts of “neuroinflammation” toward a granular map of the specific cellular players and molecular pathways involved.

While direct application of these technologies to human NPSLE brain tissue is still in its infancy, a powerful framework for understanding the disease is emerging from the synthesis of data across different platforms and models. High-resolution studies in animal models have provided critical mechanistic blueprints, identifying novel pathogenic cell subsets like choroid plexus Tfh cells and senescent DNTs. Concurrently, analyses of human CSF in related neuroinflammatory conditions have confirmed that core processes, such as intrathecal B cell clonal expansion and T cell exhaustion, are relevant features of the human CNS immune response. The integration of these findings provides a series of robust, testable hypotheses to guide future research in NPSLE.

The identification of specific cellular and molecular signatures associated with distinct clinical syndromes holds the promise of developing reliable biomarkers for diagnosis, patient stratification, and monitoring of therapeutic response. This will enable a shift from the current, syndromic classification of NPSLE to a more precise, mechanism-based stratification based on distinct “immune endophenotypes.” Such a biologically grounded framework is the essential foundation for the development of targeted therapies that can selectively neutralize the specific pathogenic pathways active in an individual patient. By bridging the gap between high-resolution molecular discovery and clinical reality, we can usher in an era of precision medicine for this severe and often devastating complication of SLE.
